# EphrinB2 induces tyrosine phosphorylation of NR2B via Src-family kinases during inflammatory hyperalgesia

**DOI:** 10.1016/j.neuroscience.2008.07.023

**Published:** 2008-09-22

**Authors:** S. Slack, A. Battaglia, V. Cibert-Goton, I. Gavazzi

**Affiliations:** Wolfson Centre for Age Related Diseases, Hodgkin Building, Wolfson Wing, King's College London, London SE1 1UL, UK

**Keywords:** EphB receptor, pain, plasticity, NMDA, Src, rat, CaMKII, calcium-calmodulin kinase II, LTP, long-term potentiation, NMDA, N-methyl-D-aspartate, PP2, 4-amino-5-(4-chlorophenyl)-7-(*t*-butyl)pyrazolo[3,4-*d*]pyrimidine, pY-NR2B, NR2B phosphorylated in tyrosine, SDS, sodium dodecyl sulfate, TBST, TBS containing 0.1% Tween-20

## Abstract

In recent years a role for EphB receptor tyrosine kinases and their ephrinB ligands in activity-dependent synaptic plasticity in the CNS has been identified. The aim of the present study was to test the hypothesis that EphB receptor activation in the adult rat spinal cord is involved in synaptic plasticity and processing of nociceptive inputs, through modulation of the function of the glutamate ionotropic receptor NMDA (N-methyl-D-aspartate). In particular, EphB receptor activation would induce phosphorylation of the NR2B subunit of the NMDA receptor by a Src family non-receptor tyrosine kinase. Intrathecal administration of ephrinB2-Fc in adult rats, which can bind to and activate EphB receptors and induce behavioral thermal hyperalgesia, led to NR2B tyrosine phosphorylation, which could be blocked by the Src family kinase inhibitor PP2. Furthermore animals pre-treated with PP2 did not develop behavioral thermal hyperalgesia following EphrinB2-Fc administration, suggesting that this pathway is functionally significant. Indeed, EphB1-Fc administration, which competes with the endogenous receptor for ephrinB2 binding and prevents behavioral allodynia and hyperalgesia in the carrageenan model of inflammation, also inhibited NR2B phosphorylation in this model. Taken together these findings support the hypothesis that EphB–ephrinB interactions play an important role in NMDA-dependent, activity-dependent synaptic plasticity in the adult spinal cord, inducing the phosphorylation of the NR2B subunit of the receptor via Src family kinases, thus contributing to chronic pain states.

Ionotropic NMDA (N-methyl-D-aspartate) receptors are well established as a key component of nociceptive signaling mechanisms in the dorsal horn of the spinal cord and are largely implicated in the establishment of central sensitization, one of the primary mechanisms responsible for development and maintenance of chronic pain (Woolf and Thompson, 1991; Ren and Dubner, 1999; Willis, 2001; Parsons, 2001; Petrenko et al., 2003; Bleakman et al., 2006). NMDA receptor activation has thus been implicated in synaptic plasticity in the dorsal horn of the spinal cord, as well as in the hippocampus (see e.g. Muller et al., 1988; Maccaferri and McBain, 1996; Berberich et al., 2007). In both systems, recent studies have revealed an important role for the NR2B subunit of this receptor as a major modulatory unit, controlling the activity of the receptor via phosphorylation mechanisms, enabling synaptic potentiation (long-term potentiation, LTP, or central sensitization) (Rosenblum et al., 1996; Guo et al., 2002). Modulation occurs through phosphorylation of the intracellular portion of NR2B at specific tyrosine residues by signal transduction proteins. In the spinal cord, studies by Guo et al. (2002) revealed that chronic inflammatory pain induced by complete Freund's adjuvant in the rat paw caused an increase in the phosphorylation state of NR2B. This could be prevented by intrathecal administration of inhibitors of the signaling kinases Src and PKC and of group I metabotropic glutamate and NK1 receptors, revealing that in this model NR2B could be modulated by glutamatergic and tachykinin nociceptive transmission, leading to hyperalgesia.

Recent studies have identified a possible role of EphB receptor tyrosine kinases as further modulators of NMDA receptor function. EphB receptors and their ephrinB ligands are transmembrane cell surface molecules, with a well-studied and important role in neural development, primarily acting as patterning and guidance cues (Wilkinson, 2001).

In the mature nervous system, several studies have reported a neuromodulatory role for EphB receptors and ephrinBs in activity-dependent synaptic plasticity in the CNS, mainly but not exclusively via an interaction with ionotropic glutamate receptors (Henderson et al., 2001; Grunwald et al., 2001; Contractor et al., 2002; Takasu et al., 2002; Grunwald et al., 2004) reviewed in Yamaguchi and Pasquale (2004) and Caló et al. (2006). We previously identified a novel role for ephrinB2 and its Eph tyrosine kinase receptor(s) *in vivo* in the spinal cord, as neuromodulators of pain signaling, probably acting via NMDA receptors (Battaglia et al., 2003).

Interestingly, Takasu et al. (2002) showed that in primary cortical neurons in culture EphB activation by ephrinB2-Fc (immunoglobulin Fc fusion protein of ephrinB2) induced Src-dependent NR2B phosphorylation and furthermore we showed that intrathecal injection of ephrinB2-Fc induces Src phosphorylation in the spinal cord of adult rats (Battaglia et al., 2003).

We therefore formulated the hypothesis that EphB receptors are involved in synaptic plasticity *in vivo* in the spinal cord (underlying chronic pain states) via a mechanism involving Src activation and NR2B phosphorylation. In the present study we tested this hypothesis using two models, ephrinB2-Fc intrathecal administration to induce pharmacological activation of the EphB receptors, and intraplantar carrageenan injection (a model of inflammation, which leads to NMDA-dependent central sensitization and behavioral hyperalgesia and allodynia, through mechanisms deemed analogous to those involved in the onset of forms of chronic pain (Eisenberg et al., 1994).

## Experimental procedures

All experiments were performed in accordance with institutional regulations and the Animals (Scientific Procedures) Act 1986. Every effort was made to minimize animal suffering, and the experiments were designed in order to use the minimum number of animals necessary to obtain valid results.

### *In vivo* application of neurochemicals

Intrathecal cannulae were implanted for delivery of the different neurochemicals used in this study in domitor (250 mg/kg) and ketamine (60 mg/kg, i.p.) -anesthetized adult male Wistar rats (Harlan, Blackthorn, Bicester, UK; 225–250 g body weight). Briefly, the mid-thoracic spinal cord was incised at the midline, muscles were separated from bone by blunt dissection, and a small laminectomy was made at the 6th or 7th thoracic vertebra. A cannula was inserted under the dura mater such that the tip rested at the lumbar enlargement. The opposite end was externalized at the top of the head. Catheters were prepared to allow for an injection volume of 10 μl of the test substance, followed by a 10 μl flush of saline solution. The application of neurochemicals took place after a minimum of 1 week in lightly restrained animals.

For the biochemical and behavioral studies investigating the effect of inhibiting Src family kinases, the rats received a bolus intrathecal injection of 10 μl 4-amino-5-(4-chlorophenyl)-7-(*t*-butyl)pyrazolo[3,4-*d*]pyrimidine (PP2, Calbiochem, San Diego, CA, USA, 73 nmol or 7.3 nmol/rat) in DMSO (10% in saline solution) or DMSO (10% in saline solution), followed by a 10 μl saline solution flush per animal.

This was followed after 5 min by a further injection with 10 μl ephrinB2-Fc solution (2 μg/rat, R&D Systems, Minneapolis, MN, USA) or saline solution (10 μl), followed by a 10 μl saline solution flush per animal.

For the biochemical studies on carrageenan-treated rats, inflammation was induced by intraplantar injection of 100 μl of lambda carrageenan (10 mg/ml, Sigma-Aldrich, Gillingham, UK) in one hind paw of restrained rats. Five minutes prior to carrageenan injection, the rats received an intrathecal injection of either EphB1-Fc (R&D Systems; 10 μg/rat in saline solution), or human IgG (2 μg/rat Fc fragment, Jackson Immunochemicals, West Grove, PA, USA), followed by 10 μl saline solution flush. Animals which had received a saline injection in the hind paw were used as controls.

EphB1-Fc and EphrinB2-Fc chimeric proteins migrate respectively as an approximately 110 kDa protein and as an approximately 60–65 kDa protein in SDS-PAGE under reducing condition.

### Behavioral studies

Catheters were chronically implanted into the lumbar subarachnoid space of anesthetized rats, as described above. Paw withdrawal latency to a radiant heat stimulus was measured using a Plantar Test apparatus (Ugo Basile, Comerio, Italy), in which plantar hind paws were exposed to a radiant heat stimulus and withdrawal latencies automatically timed. Cutoff time was 25 s. Animals were habituated to the test environment for 3–5 days prior to experimentation. On the day of testing, after acclimatization triplicate measurements of withdrawal latency were taken to provide the baseline. After administration of PP2 and EphrinB2-Fc or vehicle as described above, triplicate measurements of withdrawal latencies were taken at each time point. Paw withdrawal responses were measured at 30, 60, 90, 120 min after intrathecal injection. The experimental groups were as follows: DMSO 10% in saline-ephrinB2-Fc (R&D Systems; *n*=5), PP2 73 nmol (Calbiochem)-saline (*n*=7), PP2 73 nmol-ephrinB2-Fc (*n*=7), PP2 7.3 nmol-ephrinB2-Fc (*n*=6). Thermal hyperalgesia was defined as a significant decrease of paw withdrawal latency compared with baseline or control values.

### Biochemical studies

Forty minutes after intrathecal application of neurochemicals and/or carrageenan intraplantar injection, rats were anesthetized with pentobarbital (140 mg/kg) and the spinal cords were rapidly obtained by hydraulic extrusion. The dorsal horns of the lumbar enlargement were dissected out on ice and were then immediately snap-frozen on liquid nitrogen. They were homogenized in ice-cold lysis buffer containing protease and phosphatase inhibitors (1%v/v nonidet P-40, 2 mM EDTA, 20 mM Tris, pH 8, 137 mM NaCl, 10% v/v glycerol, 1 mM phenylmethanesulfonyl fluoride (PMSF), 5 mM NaF, 1 mM NaVO_4_, 10 μg/ml each anti-pain, leupeptin and pepstatin). The samples were left to homogenize for 2 h under rotating agitation. The extracts were centrifuged and the supernatant retained. After protein titration using a BCA protein assay (Pierce, Rockford, IL, USA) the equivalent of 500 μg of total protein was added to 5 μg of rabbit anti-NR2B (Upstate, Cambridge, UK) antibody and gently shaken overnight at 4 °C.

The next morning protein-A sepharose beads were added to the samples and gently shaken for 4 h at 4 °C. Beads were then rinsed in lysis buffer and boiled in loading buffer made of 2% sodium dodecyl sulfate (SDS), 100 mM DTT, 10% glycerol, 0.25% Bromophenol Blue for 5 min. The protein-rich supernatant was separated on an 8% acrylamide resolving gel and transferred to a PVDF membrane using a semi-dry transfer unit (BioRad, Hercules, USA). After 1 h transfer in 20% methanol transfer buffer the membranes were blocked in 5% BSA in TBS containing 0.1% Tween-20 (TBST) and were incubated overnight at room temperature in either mouse anti–phospho tyrosine (4G10) antibody (diluted 1:1000) (Upstate) or rabbit anti-phospho-Src antibody (diluted 1:1000) (Biosource, Paisley, UK), or rabbit anti phospho-tyr1472-NR2B (Imgenex, Sorrento Valley, CA, USA) or rabbit anti phospho-Ser1303-NR2B (Upstate) or rabbit anti-EphB1 (1:200, Santa Cruz Biotechnology, Santa Cruz, CA, USA). Blots were washed in TBST and incubated in peroxidase-conjugated donkey anti-rabbit IgG (1:5000, Amersham Pharmacia, Chalfont St.Giles, UK) or donkey anti-mouse IgG (1:10,000, Amersham Pharmacia) for 1 h at room temperature. All antibodies were diluted in TBST.

Protein bands were visualized using an enhanced chemi-luminescence detection kit (ECL Plus) (Amersham Pharmacia) followed by autoradiography using Hyperfilm MP (Amersham Pharmacia). The blots were then washed in TBST and stripped for 45 min in stripping buffer (2% w/v SDS, 0.8% v/v β-mercaptoethanol, 12.5% w/v Tris–HCl, pH 6.8, 0.5 M). The membranes were washed for 2 h in a continuous flow of water and blocked in 5% BSA in TBST for 1 h. Membranes were reprobed for total NR2B by overnight incubation at room temperature in rabbit anti-NR2B (Upstate). Blots were then washed, incubated for 1 h with secondary antibody (peroxidase-conjugated donkey anti-mouse IgG, 1:5000, Amersham Pharmacia) and visualized as above. Gels were scanned and captured using Adobe Photoshop (Adobe Systems Inc., San Jose, CA, USA). The densitometric analysis was made using Scion Image software (Scion Corporation, Fredrick, MD, USA) by measuring the surface area of the band (pixel squares) as well as the average pixel intensity (using a gray scale of 256 levels) for each band. Results were expressed as the level of phosphorylation for each sample i.e. as the ratio (in arbitrary units) of the phosphorylated form (phospho-tyrosine-NR2B/phospho-Src/phospho-Ser1303/phospho-Tyr1472) over total form of NR2B.

### Statistical analysis of data

Statistical analysis was performed using an ANOVA on ranks method for the analysis of bands on membranes, while two-way ANOVA and post hoc Bonferroni tests were used for behavioral data analysis. All analysis was performed using SigmaStat software (Systat Software Inc., San Jose, CA, USA). *P* values less than 0.05 were taken as significant.

## Results

### Intrathecally administered ephrinB2 induces NR2B phosphorylation via Src

We have previously shown that a single intrathecal injection of ephrinB2 could induce thermal hyperalgesia in rats within 30 min, which could be prevented by co-administration of the NMDA receptor antagonist MK801 (Battaglia et al., 2003). These data strongly suggested the involvement of this glutamate receptor in ephrinB2-mediated nociceptive processing.

**Fig. 1:**
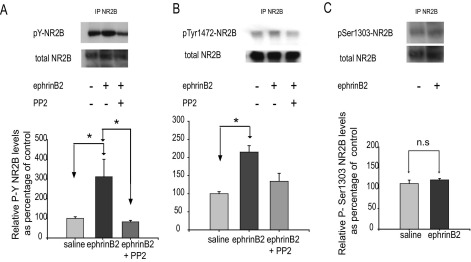
Effect of ephrinB2 treatment on phosphorylation of NR2B. NR2B was immunoprecipitated from the dorsal portion of the lumbar spinal cord of rats, which had received two intrathecal injections, separated by a 5 min interval. The first injection was either vehicle (DMSO 10% in saline, 10 μl) or the Src family kinases inhibitor PP2 (73 nmol in DMSO 10% in saline, 10 μl), and the second injection was either ephrinB2-Fc (2 μg in saline, 10 μl), or 10 μl saline solution. The state of phosphorylation of NR2B at various residues was observed in immunoblots performed using phospho-specific antibodies. (A) Representative immunoblots using anti-phosphotyrosine 4G10 (pY-NR2B) followed by anti-NR2B antibodies. Levels of tyrosine phosphorylation of NR2B were significantly increased following ephrinB2-Fc treatment (*n*=4) compared with control levels (*n*=4). This increase was prevented by prior administration of PP2 (*n*=4). (B) EphrinB2-Fc treatment induced phosphorylation of the NR2B subunit of NMDA receptor on Tyr1472. The top blot shows the immunoreactive bands against phospho-Tyr1472, while the bottom blot shows total NR2B. Tyr1472 residue was a target of phosphorylation on NR2B following ephrinB2-Fc treatment, and this phosphorylation was blocked by PP2; *n*=4 for all groups. (C) The levels of phosphorylation of NR2B on Ser1303 residue (shown in the hippocampus to be phosphorylated by CaMKII) in the lumbar spinal cord were not affected by treatment with ephrinB2-Fc. Immunoblots obtained using antibodies specific for phospho-Ser1303 (top panel) and antibodies against NR2B; *n*=3 for all groups. In all bar charts the relative phosphoprotein levels (means±S.E.M.) are expressed as percentage of the saline controls, and normalized to the respective NR2B band. In all experiments levels of total NR2B remained unchanged. Quantification carried out as described in Experimental Procedures. Test: ANOVA on ranks. * *P*<0.05.

Here we wished to investigate whether the modulatory effect of ephrinB2 on NMDA receptor could be via intracellular tyrosine phosphorylation of the NR2B subunit. Dorsal horn cord samples of saline (control) or ephrinB2 treated rats were immunoprecipitated with an NR2B subunit-specific antibody, eluted and incubated with a phospho-tyrosine specific antibody (4G10, i.e. NR2B phosphorylated in tyrosine (pY-NR2B) in Fig. 1A). The band was at the correct molecular weight for NR2B and was also recognized by a total NR2B specific antibody (Fig. 1A).

Following ephrinB2 treatment, the state of tyrosine phosphorylation of NR2B was significantly increased compared with control levels, while levels of total NR2B remained unchanged (Fig. 1A lanes 1 and 2). Administration of PP2, which inhibits the Src family of protein tyrosine kinases, prior to administration of ephrinB2, prevented the increase in NR2B tyrosine phosphorylation (Fig. 1A lane 3 vs. lane 1 and 2). PP2 alone did not alter NR2B basal phosphorylation levels (data not shown).

We further investigated whether residue Tyr1472 of NR2B could be the target of ephrinB2 signaling, by immunoprecipitating NR2B and probing with an antibody specific for the phosphorylated form of Tyr1472. In the mouse telencephalon, this residue was shown to be phosphorylated by a Src family member named Fyn (Nakazawa et al., 2001). Phosphorylation levels of Tyr1472 were significantly increased following ephrinB2 treatment (Fig. 1B), an effect which could be prevented by prior administration of PP2, suggesting a signal cascade involving Src family kinases was involved in ephrinB2 modulation of Tyr1472 phosphorylation.

It has been reported that specific serine residues on NR2B can also be modulated by protein kinases in the hippocampus: the Ser1303 residue was shown to be phosphorylated by calcium-calmodulin kinase II (CaMKII), to enable a slower dissociation of preformed CaMKII–NR2B complexes, a component of synaptic plasticity (Omkumar et al., 1996; Strack and Colbran, 1998; Strack et al., 2000). We therefore investigated whether ephrinB2 could alter the state of phosphorylation of Ser1303. Immunoprecipitation of NR2B followed by probing with the phospho-Ser1303-NR2B-specific antibody revealed no change compared with control levels (Fig. 1C). This therefore suggests that neither CaMKII nor modulation of Ser1303 phosphorylation is involved in ephrinB2 modulation of NMDA receptor activity.

### The pro-nociceptive effects of ephrinB2 are mediated by Src-family kinases

**Fig. 2:**
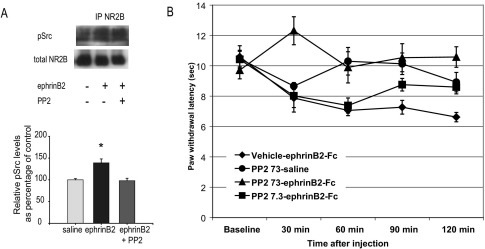
Effect of ephrinB2-Fc intrathecal treatment on the levels of phospho-Src bound to NR2B and on rat behavior in the plantar test. The animals received two consecutive intrathecal injections (separated by a 5 min interval). The first injection consisted of 10 μl of either DMSO 10% in saline or PP2 (73 nmol only for immunoprecipitation, 73 or 7.3 nmol for behavioral testing diluted in DMSO 10% saline), followed by a second injection of 10 μl of either ephrinB2-Fc (2 μg in saline) or saline only. (A) NR2B was immunoprecipitated from lysates of the dorsal horn of the lumbar spinal cord of treated rats. The top panel shows representative immunoblots performed with antibodies against phospho-Src (top) and NR2B (bottom). The levels of phospho-Src co-precipitated with NR2B were significantly increased in rats treated with ephrinB2-Fc (*n*=4) as compared with control rats (*n*=4). This increase was not present in rats pre-treated with PP2 (*n*=3). In the bar chart the relative phosphoproteins level (means±S.E.M.) are expressed as percentage of the saline controls, and normalized to the respective NR2B band. The levels of total NR2B remained unchanged. Quantification carried out as described in Experimental Procedures. Test: ANOVA on ranks. * *P*<0.05. (B) We correlated these biochemical findings with behavioral analysis. Rats were habituated to the test environment for at least 30 min; their withdrawal latency to a radiant thermal stimulus was then measured using a plantar test apparatus, to obtain a baseline level of response. The animals received then two intrathecal injections as described above. Paw withdrawal latencies were measured at 30, 60, 90 and 120 min after the injections. A significant reduction in withdrawal latency indicated the development of thermal hyperalgesia. Intrathecal PP2 treatment (73 nmol/rat) followed by saline had no significant effect on paw withdrawal latencies at any time point (ns vs. PP2-saline baseline). As expected, intrathecal ephrinB2-Fc induced thermal hyperalgesia (*P*<0.001, *n*=5; two-way ANOVA). Prior administration of PP2 prevented the effect of ephrinB2-Fc in a dose-dependent manner: animals treated with 7.3 nmol PP2 (*n*=6) displayed latencies which were intermediate between those of control (*n*=7) and ephrinB2-Fc-treated rats, with significant hyperalgesia at 60 min only (*P*<0.05), while a dose of 73 nmol totally prevented ephrinB2- induced hyperalgesia (ns. vs. control, *n*=7).

We have recently shown in the spinal cord an increase in the state of phosphorylation of non-receptor Src-family tyrosine kinases which were bound to EphB receptors “pulled-down” following ephrinB2 treatment (Battaglia et al., 2003). Furthermore, here we have shown that the Src-family kinase inhibitor PP2 could prevent ephrinB2 induction of NR2B phosphorylation. These data suggest that Src-family kinases are likely candidates for mediating the effect of ephrinB2 upon NMDA receptors. We consequently investigated the state of phosphorylation of the Tyr418 residue of Src-family kinases with identical conserved regions (Src, Fyn and Yes) co-precipitated with NR2B. Following immunoprecipitation of NR2B, membranes were probed with anti-phospho-Tyr418. We did not reprobe for amounts of total Src/Fyn/Yes as total levels of those proteins bound to NR2B are directly affected by phosphorylation, i.e. phosphorylation promotes the association of the proteins to NR2B (Yu et al., 1997); the ratio of phospho-Src/total Src could thus potentially remain constant despite a significant increase in the total amount of activated phospho-Src bound to NR2B. We therefore chose to reprobe for total levels of NR2B. Phosphorylation levels of Src-family kinases bound to NR2B were significantly elevated following ephrinB2-Fc application onto the dorsal horn (Fig. 2A).

We wished to correlate those biochemical findings with behavioral studies. As previously shown (Battaglia et al., 2003), intrathecally administered ephrinB2-Fc induced thermal hyperalgesia (Fig. 2B) (*P*<0.01, *n*=5). Prior administration of the Src-family kinase inhibitor PP2 prevented the onset of the ephrinB2-induced hyperalgesia in a dose-dependent manner: animals treated with PP2 at a dose of 7.3 nmol displayed latencies which were intermediate between those of control and ephrinB2-treated rats (*n*=6), and significantly lower than control latencies, however only at 60 min (Fig. 2B), while a dose of 73 nmol totally prevented the hyperalgesia induced by ephrinB2 (latencies after injection not significantly different from control rats and baseline, *n*=7) (Fig. 2B). Control rats (injected with PP2 73 nmol followed by saline) remained unchanged throughout the experiment (all time points not significantly different from baseline, Fig. 2B) (*n*=7).

### Sequestration of ephrinB2 following chronic inflammatory pain in the cord prevents the rise in NR2B phosphorylation

**Fig. 3:**
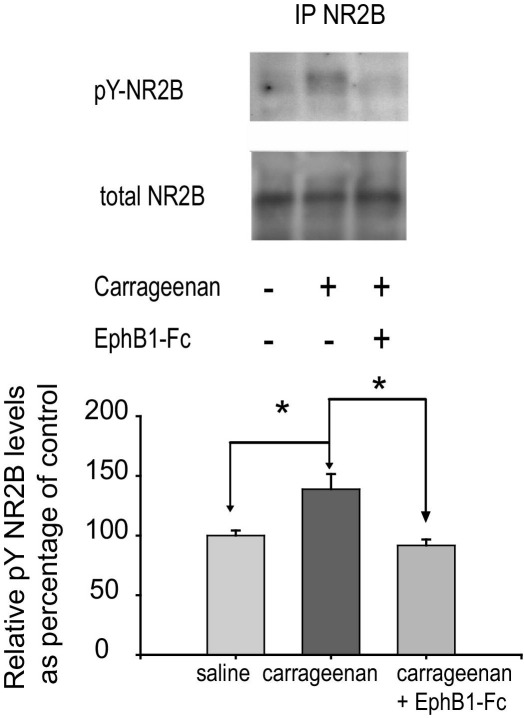
Intrathecal administration of EphB1-Fc can prevent the increase in tyrosine phosphorylation of NR2B induced by intraplantar injection with carrageenan. Rats received an intraplantar injection of carrageenan (1 mg/ml, 100 μl), preceded by intrathecal injection with either EphB1-Fc (10 μg/rat in saline, 10 μl) or Human-Fc in saline (2 μg/rat in 10 μl). After 40 min the lumbar portion of their spinal cords was dissected, and NR2B was immunoprecipitated from the dorsal portion of the cords. The top panel shows representative immunoblots using anti-phosphotyrosine 4G10 (pY-NR2B) and anti-NR2B antibodies. Forty minutes following the noxious stimulation induced by carrageenan injection there was a marked and significant increase in the state phosphorylation of NR2B (pY-NR2B). Intrathecal administration of the soluble Eph-B1 receptor chimera prior to the carrageenan injection significantly reduced the phosphorylation levels of NR2B. Levels of total NR2B remained unchanged. This suggests that the endogenous ephrin in the dorsal horn was prevented from binding to EphBs in the spinal cord by the soluble receptor, hence preventing the phosphorylation of NR2B induced by carrageenan. In the bar chart the relative phosphoproteins level (means±S.E.M.) are expressed as percentage of the saline controls, and normalized to the respective NR2B band. Quantification carried out as described in Experimental Procedures. Test: ANOVA on ranks. * *P*<0.05.

The above biochemical and behavioral findings provide information into the role of exogenously applied ephrinB2-Fc. A physiological model of nociception was indispensable to validate these data and a model of chronic inflammatory nociception induced by injecting carrageenan in the hind paw was chosen. Forty minutes following the noxious stimulation induced by carrageenan injection a marked and significant increase in the state of pY-NR2B was observed in the lumbar area of the dorsal horn of the cord (Fig. 3). Levels of total NR2B remained unchanged. Intrathecal administration of the soluble Eph-B1 receptor chimera prior to the carrageenan injection significantly reduced the phosphorylation levels of NR2B (Fig. 3). This suggests that the interaction of endogenous ephrin with EphB receptors on dorsal horn neurons was prevented by the soluble receptor, hence preventing the phosphorylation of NR2B induced by carrageenan.

### NMDA and EphB1 receptor are present in the same complex

**Fig. 4:**
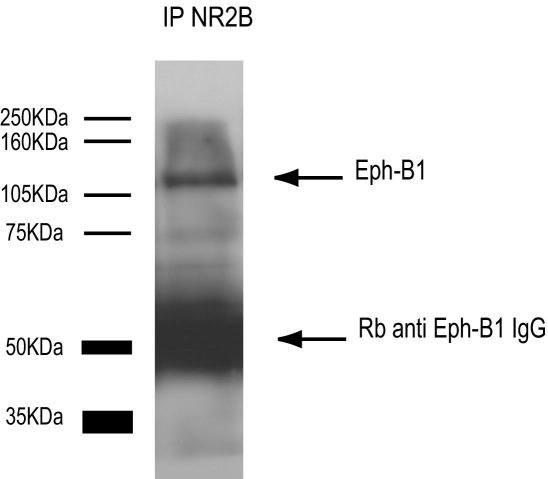
NR2B and EphB1 receptors co-immunoprecipitate. The lumbar region of the spinal cord of naive rats was dissected, and NR2B was immunoprecipitated. Representative immunoblots obtained using antibodies against EphB1 receptor. We were able to co-immunoprecipitate Eph-B1 with NR2B (*n*=5). We can therefore conclude that the two receptors are in close association in the membrane of dorsal horn neurons.

We previously demonstrated that ephrinB2 induces a hyperalgesic response and that carrageenan-induced hyperalgesia could be reduced with a soluble Eph-B1 chimera, which, however, due to the promiscuous binding of EphB receptors, could block the activation by endogenous ephrinB2 of any EphB receptor present in the spinal cord. It remained therefore to be determined which Eph-B receptor was mediating those effects. Eph-B1 is present in the adult spinal cord dorsal horn neurons (Battaglia et al., 2003), Eph-B2 is found in meningeal fibroblasts of the spinal cord (Bundesen et al., 2003), while Eph-B3 is found in low amount in normal animals, but is upregulated in white matter astrocytes and motoneurons following a spinal cord injury (Miranda et al., 1999). We therefore here investigated whether Eph-B1 could be the receptor responsible for ephrinB2 signaling to NR2B. We were able to co-immunoprecipitate Eph-B1 with NR2B (Fig. 4). We can therefore conclude that the two receptors are at least in close association in the membrane of dorsal horn neurons.

## Discussion

The role of the NMDA receptor in the phenomenon of activity-dependent synaptic plasticity is well established, and NMDA-mediated synaptic plasticity has been shown to be essential for the development of chronic inflammatory pain and neuropathic pain (see e.g. Petrenko et al., 2003). NMDA receptor function can be modulated by phosphorylation mechanisms (and in particular tyrosine phosphorylation, Ali and Salter, 2001; Salter and Kalia, 2004) by neighboring activated receptors including TrkB, mGluRs and the NMDA receptor itself (Woolf and Salter, 2000; Guo et al., 2004; Mantyh and Hunt, 2004), both in the hippocampus and in the spinal cord (Rosenblum et al., 1996; Guo et al., 2002). However, the relative importance of the different pathways, and the different role of the multiple NMDA receptor subunits, is still controversial, and it is not clear if the same mechanism is involved in the different forms of chronic pain. Studies in recent years (reviewed in Gogas, 2006) have focused on the role of the NR2B subunit of the receptor, which is important for receptor localization and endocytosis, in pathological pain states. There are some findings (Guo et al., 2002; Abe et al., 2005) which suggest a role for NR2B tyrosine phosphorylation in inflammatory and neuropathic pain (even if the role of NR2B specific antagonists in analgesia has also been attributed to supraspinal, rather than intraspinal effects).

Here we provide evidence for the role of a further receptor, the EphB receptor, in controlling the phosphorylation of NMDA receptor in the spinal cord in vivo, acting on the NR2B subunit. We had previous evidence for a role for the EphB receptor in synaptic plasticity *in vivo* in the spinal cord, which we proposed could be mediated by NMDA receptors (Battaglia et al., 2003). Immunohistochemical studies revealed expression of EphB1 receptors in neurons in the dorsal horn and of the ligand ephrinB2 on small nociceptive neurons (both at the level of the cell body and on terminals in the dorsal horn). Activation of EphB receptor via intrathecal injection of EphrinB2-Fc chimeras induced behavioral thermal hyperalgesia in freely moving rats, which could be counteracted by prior injection of the non-competitive NMDA receptor antagonist MK801, suggesting an involvement of the NMDA receptor. The mechanism apparently involved Src family kinases, since the level of phospho-Src bound to EphB receptors in the dorsal horns of the spinal cord was increased. Conversely, blockage of EphB receptor activation by endogenous ephrin via injection of EphB1-Fc chimeras was sufficient to inhibit the onset of thermal hyperalgesia and mechanical allodynia in a model of inflammatory pain (carrageenan injection), onset which is presumed to require sensitization of neurons in the dorsal horn, and significantly reduced pain-related behavior in the second phase of the formalin test (a model of chemically induced pain), which again requires central sensitization. The increase in c-fos expression in the superficial laminae of the spinal cord in this model was also inhibited.

In the present study we provide more direct evidence supporting the hypothesis of a role of EphB receptors in physiological synaptic plasticity *in vivo*, via modulation of NMDA receptor activity, and in particular via Src-mediated phosphorylation of the NR2B subunit. We first showed that intrathecal injection of ephrinB2-Fc chimeras, which induces thermal hyperalgesia, also induced NR2B phosphorylation on one or more tyrosine residues, including tyrosine 1472. The Tyr1472 residue was shown to be phosphorylated *in vivo* in the hippocampus following LTP, confirming a role for this residue in synaptic plasticity (Nakazawa et al., 2001). Furthermore phosphorylation of Tyr1472 seems crucial for the maintenance of neuropathic pain (Abe et al., 2005) and is one of the residues required for EphB2-mediated NMDA receptor opening in cultured cortical neurons (Takasu et al., 2002), indicating a possible mechanism for the modulatory effect of tyrosine phosphorylation on receptor function. Both the behavioral thermal hyperalgesia and NR2B phosphorylation were inhibited in a dose-dependent manner in the present work by preceding ephrinB2-Fc injection with injection of the Src family inhibitor PP2. We also showed that blockage of endogenous EphB receptor activation by intrathecal injection with EphB1-Fc chimeras, which we had previously shown to inhibit the behavioral and thermal hyperalgesia induced by carrageenan injection in the hind paw (Battaglia et al., 2003), also inhibited the phosphorylation of NR2B induced by this injection. It must be noted that Caudle et al. (2005) did not observe an increase in NR2B phosphorylation in carrageenan-treated rats, in contrast to our present findings in the same model, and those obtained by Guo et al. (2002) after complete Freund's adjuvant or mustard oil application. The reasons for this discrepancy are not clear. The most likely explanation is a difference in the time points examined, and possibly in the protocol used to quantify NR2B phosphorylation.

Previous studies (extensively reviewed in Caló et al., 2006) had suggested a role for the EphB2 receptor in modulating the function of NMDA receptor in the hippocampus, affecting both LTP and LDP, analogous to the role we now propose for Eph receptors in the spinal cord. However, these studies (Henderson et al., 2001; Grunwald et al., 2001) were conducted in transgenic mice, therefore developmental defect could not be excluded, and the mechanism of action *in vivo* could not be identified with certainty. Takasu et al. (2002), working with cultured embryonic cortical neurons, established that EphB receptor activation in these neurons, obtained with treatment with ephrinB2-Fc chimeras, induced Src family kinases phosphorylation at residue Tyr416, with consequent NR2B receptor phosphorylation and increased Ca^2+^ influx through NMDA receptors upon glutamate administration. They also showed that in HEK293T cells mutation of the Src-binding site on EphB2 or expression of a dominant negative of Fyn (inhibiting Src family kinases) disabled the phosphorylation of NR2B effect of ephrinB2 and the potentiation of NMDA receptor activity.

Both our present findings and those by Guo et al. (2002) suggest that, in analogy to what shown by Takasu et al. (2002) in cultured embryonic neurons, a Src family kinase is responsible for the tyrosine phosphorylation of NR2B in inflammatory pain. Evidence exists for a role at least of Fyn and Src in NMDA receptor phosphorylation (Salter and Kalia, 2004), therefore we cannot exclude that a combination of at least these two Src family kinases is responsible for ephrinB2-mediated NR2B phosphorylation in our spinal cord plasticity model. Nakazawa et al. (2001) reported 25 tyrosine residues at the carboxyl tail of NR2B which could be phosphorylated by Fyn, including Tyr1252, Tyr1336, and Tyr1472 residues. We do not know how many of these residues are phosphorylated in our model, further to Tyr1472. For an extensive review on the role of Src family kinases in NMDA-dependent synaptic plasticity, see (Kalia et al., 2004).

Other subunits of the NMDA receptor may be targeted by Src-family kinases (Ali and Salter, 2001). Indeed, EphB2 activation of Src by phosphorylation in cultured hippocampal and cortical neurons could induce NR2A phosphorylation (Grunwald et al., 2001). However NR2A phosphorylation was unchanged following complete Freund's adjuvant-induced noxious stimulation, suggesting it is unlikely that this subunit has a role in hyperalgesia (Guo et al., 2002). NR2A and NR2B subunits seem to have different distribution in the spinal cord, and this may reflect a difference in function (Nagy et al., 2004).

In the present study we have exclusively investigated the interaction between EphB receptor activation and NMDA phosphorylation. We cannot exclude a contribution of NMDA receptor regulation via mechanisms other than tyrosine phosphorylation. Furthermore, we cannot exclude the possibility that EphB receptor activation may regulate the function of other receptors, further to the NMDA receptor. EphB receptors could potentially regulate AMPA receptor trafficking (reviewed in Caló et al., 2006).

An unavoidable limitation of the current study is that we cannot incontrovertibly identify the specific EphB receptor responsible for the effect we observed, due to the lack of appropriate tools to activate or inhibit individual EphB receptors selectively. Here we show that EphB1 and NMDA receptors co-immunoprecipitate in dorsal spinal cord preparation, indicating that they are associated in a complex, even if we do not know if they are directly physically associated, as shown by Dalva et al. (2000) for EphB1-B4 and the NR1 subunit of NMDA receptors in transfected 293T cells and cultured hippocampal neurons. Our current finding, together with our previous immunohistochemical localization data, and studies performed by us on EphB1 knockout mice, published only in abstract form (Cibert-Goton et al., 2005, 2006), support the hypothesis that EphB1 receptors are indeed involved in controlling synaptic plasticity at spinal cord level, possibly playing the same role that EphB2 plays in higher centers.

It remains to be determined whether the same mechanisms are also responsible for other chronic forms of hyperalgesia, such as those associated with nerve injury. A role for EphB1-ephrinB2 interactions in neuropathic pain has recently been identified, providing further support to the hypothesis that this receptor is the main contributor to synaptic plasticity of excitatory synapses in the spinal cord (Kobayashi et al., 2007; Song et al., 2008a,b). Kobayashi et al. (2007) found that expression of ephrinB2 in dorsal root ganglion neurons was enhanced by nerve injury, and EphB1 receptor expression was observed in the dorsal horn of the spinal cord of adult rat. Administration of ephrinB2 siRNA decreased expression of ephrinB2 and mechanical allodynia after spinal nerve crush. Song et al. (2008a) studied the expression of ephrinB1 and EphB1 in the dorsal root ganglia and spinal cord after chronic constriction injury (a model of neuropathic pain), dorsal rhizotomy or a combination of both. Their findings, including a significant upregulation of ephrinB1 and EphB1 in the spinal cord and dorsal root ganglia of adult rat after chronic constriction injury, which was time dependent and correlated with the development of thermal hyperalgesia, support the hypothesis of an involvement of this system in the onset of neuropathic pain.

A very recent article by Song et al. (2008b), made available online at the time of submission of the present work, adds further compelling support to the hypothesis of an involvement of EphB receptor-ephrinB signaling in both onset and maintenance of neuropathic pain. These authors found that EphB1-Fc and EphB2-Fc chimeras, used as blocking agents for EphB receptor activation, prevented the onset of mechanical allodynia and thermal hyperalgesia, and transiently reversed established allodynia and hyperalgesia, in the chronic constriction injury model of neuropathic pain. This behavioral observation correlated with parallel electrophysiological findings, that EphB-Fc administration prevented hyperexcitability of nociceptive neurons in the dorsal root ganglion and sensitization of wide dynamic range neurons in the dorsal horn in neuropathic rats. In the same model, activation of EphB receptors with ephrinB-Fc in isolated dorsal root ganglia induced hyperexcitability of small nociceptive neurons, but only in injured animals. Interestingly, the authors also found that activation of EphB receptors promotes LTP of synapses between nociceptive dorsal root ganglion neurons and dorsal horn neurons in intact rats: this mechanism could also contribute to the role of the EphB-ephrinB system in inflammatory pain.

The evidence presented here, together with previously reported findings described above (Battaglia et al., 2003; Kobayashi et al., 2007; Song et al., 2008a,b) would suggest that the interaction of presynaptic ephrinB2 (and in neuropathic pain also possibly ephrinB1) with EphB receptors is required for the onset of different forms of persistent pain (both inflammatory and neuropathic). Our findings (Battaglia et al., 2003, and present work) support the idea of an activation by ephrinB2 of postsynaptic EphB receptors in neurons of the dorsal horn, since we showed that EphB1 receptors are present in dorsal horn neurons, where they are found in close association with NMDA receptors, and intrathecal administration of ephrinB2-Fc induces an increase in phosphorylated Src kinase associated with the EphB receptor itself. Src phosphorylation is required for phosphorylation of the NR2B subunit of the NMDA receptor and for induction of thermal hyperalgesia by ephrinB2. The mechanism we proposed for a physiological role of ephrinB2 in inflammatory hyperalgesia and allodynia (Battaglia et al., 2003) was a rapid increase in the expression of ephrinB2 in the presynaptic membrane, with consequent clustering and activation of EphB receptors postsynaptically. The study by Kobayashi et al. (2007) and Song et al. (2008a,b) would suggest that in more chronic forms of pain this may be accompanied by an increase in synthesis of the ephrinB molecule. The article by Song et al. (2008b) introduces the interesting possibility of a presynaptic site of action for EphB receptor activation, which however seems to require injury of dorsal root ganglion neurons, followed by upregulation of the EphB1 receptor in the ganglion. We found no evidence of expression of EphB1 in small nociceptive neurons in intact rats (Battaglia et al., 2003); it is therefore possible that EphB activation in nociceptive neurons occurs only after nerve injury. However, binding of ephrinB2 on nociceptive neurons to EphB1 in dorsal horn neurons could itself trigger reverse signaling in nociceptors, since ephrinB2 activation can activate intracellular signaling cascades, and in other systems this form of retrograde signaling has been shown to regulate synaptic activity (reviewed in Caló et al., 2006). While activation of ephrinB2 by EphB1 in intact animals does not appear to occur, or if it does occur it appears to have no functional consequences, since we (Battaglia et al., 2003) observed no change in thermal and mechanical sensitivity after intrathecal administration of EphB1 in control rats, it is possible that in animals sensitized by inflammation or injury retrograde signaling in nociceptors does take place. Our findings do not allow us to either confirm or exclude this possibility.

## Conclusion

In conclusion, the findings presented here add to our understanding of the function of the EphB receptors–ephrin system in the adult nervous system *in vivo* (Yamaguchi and Pasquale, 2004), as important players in activity-dependent synaptic plasticity, acting as modulators of NMDA receptor function *in vivo* in the spinal cord. Furthermore, our results contribute to develop our understanding of the mechanisms underlying the phenomenon of central sensitization in the spinal cord, which has been hypothesized to be necessary for the onset and maintenance of forms of chronic pain, supporting the view of a role for phosphorylation of spinal NR2B in inflammatory pain, first shown by Guo et al. (2002).
